# Social-Ecological Resilience Moderates the Effectiveness of Avoidant Coping in Children Exposed to Adversity: An Exploratory Study in Lithuania

**DOI:** 10.3389/fpsyg.2020.536353

**Published:** 2020-10-07

**Authors:** Francesca Giordano, Simona C. S. Caravita, Philip Jefferies

**Affiliations:** ^1^Department of Psychology, Catholic University of the Sacred Heart, Milan, Italy; ^2^Norwegian Centre for Learning Environment and Behavioural Research in Education, University of Stavanger, Stavanger, Norway; ^3^Department of Psychology, Catholic University of the Sacred Heart, Brescia, Italy; ^4^Resilience Research Centre, Faculty of Health, Dalhousie University, Halifax, NS, Canada

**Keywords:** coping strategies, social ecological resilience, resilience (psychological), depression – psychology, children “with difficulties”, child trauma

## Abstract

**Background:**

Against the high prevalence of adverse childhood experiences in Lithuania, the government testified a lack of effective ways to address the problem. A crucial endeavor for intervention planners is to identify the risk and protective factors whose interaction may lead at risk children to achieve greater levels of functioning. Internal qualities and external resources can act independently or interactively to reduce the damaging effects of adversities, and to enhance resilience process. In particular, both coping strategies and social resources have been shown to have a consistent influence on trauma-related outcomes.

**Objective:**

The aim of this study was to investigate the potential interaction of coping strategies with external resources in predicting trauma-related outcomes in children exposed to adversities.

**Participants and Setting:**

A sample of 372 Lithuanian children (mean age = 13.03; range: 7–17) with a history of traumatic experiences has been involved.

**Methods:**

The Child and Youth Resilience Measure-Revised (CYRM-R), the Children Coping Strategy Checklist (CCSC), and the Trauma Symptom Checklist for Children (TSCC) have been administered to participants. A moderation analysis was performed to test whether social-ecological resilience moderate the relationship between each coping strategy and trauma-related outcomes. Results: When controlling for sex, age, other coping strategies, and social-ecological resilience, only active coping was found to significantly predict each of the trauma-related symptoms. Furthermore, social-ecological resilience has a negatively moderating effect on the relationship between avoidant coping strategies and depression.

**Conclusion:**

MHPSS professionals who design and implement interventions to enhance the likelihood of resilience among vulnerable children, should take in considerations the multiple interaction between social-ecological resilience and avoidant coping strategies in the children adjustment.

## Introduction

Within the World Health Organization’s (WHO’s) European Region, levels of adverse childhood experiences appear to be higher in the east countries than in the west ones (Sethi et al., 2013). The high burden of adverse childhood experiences and the potential cost–effectiveness of their prevention make a compelling argument for increased investment in the prevention of such experiences and for mainstreaming such prevention into many areas of health and social policy (Bellis et al., 2014). In particular, the Lithuanian children’s rights ombudsman testified a lack of effective ways to address the problem, and this led to governmental support for a new children’s support center to provide special care for children (Lithuania Human Rights Report, 2016).

The negative consequences of adverse childhood experiences are numerous and well-reported in literature, being strongly associated with externalizing and internalizing problems (Manly et al., 2001; Litrownik et al., 2005; Alisic et al., 2014), psychiatric diagnoses (Rutter, 2006; Giordano et al., 2012), impairments in cognitive functioning (Liaw and Brooks-Gunn, 1994), a reduced sense of mastery (Giordano et al., 2015), and difficulties with peers (Kelly et al., 2015). The three more widely studied adverse outcomes in children exposed to trauma are PTSD, depression and anxiety (Pine and Cohen, 2002). Some studies indicate that gender and age can influence the reactions of children who are exposed to traumatic experiences, with poorer adjustment for girls (Feiring et al., 1999; Olff et al., 2007) and for younger age groups (Lonigan et al., 1991; Vizek-Vidović et al., 2000), while others found no systematic gender and age differences on various trauma-related outcomes (Green et al., 1991; Tolin and Foa, 2006; Maikovich et al., 2009).

However, adverse childhood experiences do not automatically lead to adverse consequences; children exposed to the same type of adversity may react differently, and achieve “resilient” outcomes (Cicchetti, 2013; Domhardt et al., 2015; Ben-David and Jonson-Reid, 2017). Resilience may be thought of as a universal capacity which allows a person, group or community to prevent, minimize or overcome the damaging effect of adversity (Grotberg, 1995). Several studies have shown that resilience is consistently associated with positive trauma-related outcomes (Masten and Coatsworth, 1998; Luthar and Cicchetti, 2000; Luthar et al., 2000; Masten, 2001; Herrenkohl, 2011). In particular, it is associated with adaptive outcomes among children who are victims of violence (Ellenbogen et al., 2014; Giordano et al., 2018) and other trauma (Wolmer et al., 2011; Baum et al., 2013; Fu et al., 2013; Sanderson et al., 2016).

While resilience has been traditionally thought of as a psychological trait, more recently it has been conceptualized as a dynamic process that involves drawing on both internal and external resources to achieve positive outcomes despite adversity (Masten, 2001; Bonanno, 2012; Sanders et al., 2015; Cesana et al., 2018; Giordano and Ferrari, 2018). Ungar (2008) has accounted for this use of internal and external resources in his definition of resilience, which he describes as “both the capacity of individuals to navigate their way to the psychological, social, cultural, and physical resources that sustain their wellbeing, and their capacity individually and collectively to negotiate for these resources to be provided and experienced in culturally meaningful ways” (p. 225). While many studies of resilience have focused on internal resources (Block and Kremen, 1996; Hu et al., 2015), more are beginning to consider the importance of “social-ecological resilience,” or the importance of resources around and available to individuals (Ungar, 2008; Ungar et al., 2013). This broader understanding of resilience, which foregrounds the use and availability of internal and external resources (collectively considered “protective factors”) is used in this study.

A crucial endeavor for intervention planners is to identify the risk and protective factors – both internal and external – whose interaction may lead at-risk children to achieve greater levels of functioning (Happer et al., 2017). These factors may orient the Mental Health and Psychosocial Support (MHPSS) professionals’ actions and, as a result, lead to successful interventions aimed to strengthen them. In this regard, individual coping strategies have been shown to have a consistent influence on trauma-related outcomes (Runtz and Schallow, 1997; Tremblay et al., 1999; Kraaij et al., 2003; Flett et al., 2012) in children exposed to adversity.

Coping has been defined as the sum of constantly changing cognitive and behavioral efforts to manage specific external and/or internal demands that are appraised as taxing or exceeding the resources of the person (Lazarus and Folkman, 1984). It is an umbrella term and traditionally authors (e.g., Lazarus, 1993; Compas et al., 2001) distinguish between problem-focused coping (efforts directed toward the stressor presented by the environment), emotion-focused coping (efforts directed toward the negative emotions consequential to stress), and avoidance coping (efforts directed toward the minimization/denial of the stressor) (Endler and Parker, 1990). Nevertheless, recent studies advise to focus on the effect of specific types of coping strategies, rather than using general categories (i.e., problem-focused vs. emotion-focused) that may fail to convey the multidimensional nature of coping (Skinner et al., 2003). In this regard, Ayers and Sandler (1999) proposed a four-factor model of coping strategies: *active coping* (efforts directed toward the stressor presented by the environment, by means of problem-solving behaviors and a cognitive restructuring of the situation), *distraction* coping (efforts directed toward distracting from the stressors and physical release of emotions), *seeking social support* (efforts directed toward seeking support from others to front the stressor situation) and *avoidance coping* (efforts directed toward the minimization/denial of the stressor). This conceptualization is used in this study.

The adaptive or maladaptive nature of each coping strategy is not entirely clear, which may be due to differences in the conceptualization and measurement of the coping construct (Compas et al., 2001). Some authors state that emotion-focused coping positively correlates with anxiety, symptoms of depression (Sesar et al., 2010), emotional instability, and general maladjustment (Carlo et al., 2012), even though both positive behaviors, like emotional expressiveness, and negative behaviors, including distraction strategies such as denial and substance abuse (Whiffen and Macintosh, 2005), are included within emotion-focused coping strategies. Support seeking coping strategies are often associated with child wellbeing (Dumont and Provost, 1999).

Notwithstanding some evidence has been found of lower anxiety and depression symptoms, and fewer risk behaviors such as unprotected sexual intercourse and substance abuse associated with avoidance strategies (Dashora et al., 2011), a greater consensus seems to indicate that avoidance strategies are associated with higher levels of internalizing symptoms (Guerra et al., 2016), negative outcomes (Kraaij et al., 2003; Flett et al., 2012), low clinical compliance, and therapy dropout (Briere and Scott, 2006). Adverse childhood experiences and exposure to stressors in early life are usually associated with high levels of avoidance coping and low levels of active coping (Bal et al., 2003; Taylor and Stanton, 2007; Shikai et al., 2008; Min et al., 2013). A wide range of psychological interventions for the treatment and prevention of psychopathology have been designed to enhance the coping skills of children and adolescents (e.g., Clarke et al., 1995; Kendall et al., 1997; Chandler et al., 2015). In particular, several evidence-based psychological treatments for trauma related disorders in childhood and adolescence (e.g., the Coping with Accident Reaction (CARE) intervention group) provide children and parents with general coping strategies to prevent or manage parents and child distress (De Young et al., 2016).

At the same time, social-ecological aspects of resilience are considered to be external protective factors in the adjustment of at risk children: whether formal or informal, supportive relations have been shown to exert a remarkable effect on outcomes for youth in at-risk situations (Sanders et al., 2017; Lou et al., 2018). In particular, supportive parental interactions with the child and extended social support (Benzies and Mychasiuk, 2009; Bhana and Bachoo, 2011), spirituality and a sense of connectedness within the community (Black and Lobo, 2008), positive peer relationships (Benzies and Mychasiuk, 2009), availability of professionally administered psychosocial support (Vermeulen and Greef, 2015), and good schools (Amatea et al., 2006; Bhana and Bachoo, 2011) have been implicated in positive outcomes in populations exposed to adversities.

Internal qualities and external resources can act independently or interactively, intensely or moderately, singly or in combination, to prevent, reduce or overcome the damaging effects of adversities, and to contribute to the enhancement and/or transformation of lives (Grotberg, 1995). Some programs aimed at promoting resilience in children and adolescents exposed to adversities have been designed to enhance in parallel positive coping skills and social resources (Ayers et al., 2014; Chandler et al., 2015; Jordans et al., 2016). However, while some studies have examined the interaction between coping skills and other individual protective factors such as ego-resiliency (Menesini and Fonzi, 2005) and upward social comparisons (Hooberman et al., 2010), to our knowledge no research has investigated the potential interaction of coping strategies with external resources in predicting trauma-related outcomes in children exposed to adversities. We addressed this gap and hypothesized that:

(1) Coping strategies will be associated with trauma-related symptoms. In particular we hypothesize that higher levels of avoidance and distraction strategies will be associated with higher levels of trauma related symptoms, while higher levels of active coping and support seeking strategies will be associated with lower levels of trauma-related symptoms.
(2) There will be a negative association between measures of social-ecological resilience and trauma-related symptoms. That is, higher levels of social-ecological resilience will be associated with lower levels of trauma-related symptoms.
(3) There will be significant interactions between levels of social-ecological resilience and coping strategies as they predict levels of trauma-related symptoms. Based on the positive effects of social-ecological resilience in improving adjustment to adverse experiences, we hypothesize that social-ecological resilience can moderate the effects of the associations between different coping strategies and trauma-related outcomes, by lowering possible negative effects of avoidance and distraction and strengthening the positive effect of active coping and support seeking strategies.

## Materials and Methods

### Participants

A sample of 372 Lithuanian children (49% female) aged 7–17 (*M* = 11.93; *SD* = 3.07) participated in the study. According to the distinction assumed for the CYRM questionnaire (see later), 42.7 % of the children were aged 7 to 11 and 57.3% were aged 12–17. All participants had experienced some form of prior trauma and more than half had experienced multiple kinds (51.1%), including: emotional abuse (30.1%), domestic violence (18.0%), physical abuse (8.9%), neglect (6.2%), educational abuse (3.8%), sexual abuse (1.9%), and other kinds of trauma (26.6%; e.g., being in a serious car accident). Details about sample characteristics by traumatic experiences are reported in Table 1.

**TABLE 1:** Sample characteristics by traumatic experience (% of group).

	**Emotional abuse**	**Domestic violence**	**Physical abuse**	**Neglect**	**Educational abuse**	**Sexual abuse**	Other trauma**
Males (*n* = 177)	57 (32.2)	33 (18.6)	15 (8.5)	9 (5.1)	6 (3.4)	1 (.6)	46 (26.0)
7–11 years (*n* = 85)	32 (37.6)	17 (20.0)	4 (4.7)	4 (4.7)	4 (4.7)	0 (0.0)	28 (32.9)
12–17 years (*n* = 89)	25(28.1)	16(18.0)	11(12.4)	5(5.6)	2(2.2)	1(1.1)	18(20.2)
Females (*n* = 182)	53 (29.1)	33 (18.1)	18 (9.9)	13 (7.1)	8 (4.4)	6 (3.3)	51 (28.0)
7–11 years (*n* = 74)	16 (21.6)	12 (16.2)	4 (5.4)	4 (5.4)	2 (2.7)	1 (1.4)	25 (33.8)
12–17 years (*n* = 106)	37 (34.9)	21 (19.8)	14 (13.2)	9 (8.5)	6 (5.7)	5 (4.7)	26 (24.5)
Total* (*n* = 372)	112 (30.1)	67 (18)	33 (8.9)	23 (6.2)	14 (3.8)	7 (1.9)	99 (26.6)

*A total of 13 individuals did not report being male or female and 10 did not report their age. **Other trauma includes incidents such as being in a serious car accident.

Participants were invited to take part in the study if they had a history of any of the above traumatic experiences, but were excluded if they were experiencing acute psychosis, cognitive impairments, developmental disorders, and severe conduct disorders, as these may have impaired their ability to complete the assessments.

### Procedure

Participants were referred to the study by schools from high risk poverty areas/located in high-risk neighborhoods or day-care centers located in the following regions of Lithuania: Alytus (Lazdijai); Marijampolè (Kalvarija; Marijampolè, Šakiai); Tauragè (Jurbarkas); Kaunas (Kaišiadorys; Jonava; Garliava; Kaunas); Vilnius (Vilnius; Trakai); Utena (Utena; Molètai); Panevėžys (Ramygala; Pasvalys); Šiauliu (Šiauliai; Joniskis); and Klaipėda (Klaipėda).

The administration of the study assessment tools was conducted by a network of 31 therapists, spread across the different regions, and who specialized in the assistance of children who experienced violence. This network was coordinated by the “Paramos Vaikams Centras^1^”, a non-governmental organization founded in 1995, specialized in assistance to children and families exposed to violence, and conduct child protection programs all over Lithuania.

Informed consent was required for all participants and was provided by their caregivers or other legal guardians after a short presentation about the study. Caregivers and guardians were informed that participation could be declined without consequence. None of the participants opted to withdraw from the research.

This study is related to an international 4-year child protection program run by the Bureau International Catholique de l’Enfance (BICE) in partnership with the OAK Foundation. The BICE commissioned the Resilience Research Unit (RiRes) of the Catholic University of the Sacred Heart of Milan to conduct a study of the resilience of Lithuanian children exposed to adversity, in addition to the training program of the Assisted Resilience Approach Therapy (ARAT), delivered to a team of professional psychotherapists assisting children exposed to different kinds of adversity in 25 day-care centers across Lithuania (Giordano et al., 2018, 2019b). The study was reviewed and approved by the Scientific Committee of the Department of Psychology – Resilience Research Unit (RiRes) of the Università Cattolica del Sacro Cuore of Milan.

### Design and Measures

To address the hypotheses of the study, a brief self-report survey was compiled using validated measures of trauma, resilience, and coping strategies. We used the *Trauma Symptom Checklist for Children* (TSCC; α = 0.81–0.88; Briere, 1996) to assess traumatic symptoms. The measure consists of 54 items that explore self-reported levels of trauma-related symptoms in children and adolescents involved in traumatic experiences. It includes six clinical scales: Anxiety, Depression, Post-Traumatic Stress, Dissociation, Anger, and Sexual Concerns. The broad range of forms of childhood adversity that the study sample was exposed to led us to consider that it would be prudent to focus on just the three more widely studied adverse outcomes following trauma – PTSD, depression and anxiety (Pine and Cohen, 2002). This meant omitting the Anger, Sexual Concerns and Dissociation scales, which are more useful in the contexts of major traumatic events (e.g., physical or sexual abuse, major loss, or witnessing violence). Each item represents a specific symptom and is rated on a four-point Likert scale expressing how often the symptom is experienced, ranging from 0 (“never”) to 3 (“almost all of the time”). Example items include “worrying about things” for the Anxiety scale, “feeling sad or unhappy” for the Depression scale, and “scary ideas or pictures just pop into my head” for the PTSD scale. Scores range from 0 to 30 for the PTS and from 0 to 27 for the Depression and Anxiety scales, where higher scores indicate greater levels of experienced symptomatology.

The child and youth resilience measure-revised (CYRM-R; α = 0.82; Jefferies et al., 2018) is a 17-item self-report questionnaire designed to assess an individual’s level of social-ecological resilience, by assessing the availability and accessibility of external resilience resources (Ungar, 2011; Ungar et al., 2013). Example items include: “I feel supported by my friends” and “feel safe when I am with my family/caregiver(s).” For each statement, participants use a 3-point Likert scale ranging from 0 to 2 to express their agreement. Total scores range from 0 to 34, with higher scores indicating greater resilience. The measure was originally designed for individuals aged 10–23, but a version for ages 5–9 is available^2^, which includes simpler wording. In this study, children aged 7–11 completed the younger child version with the simpler wording, while those aged 12–17 completed the standard version. As part of initial exploratory data analysis, we confirmed the equivalence of the measures by comparing scores of individuals who completed the younger child version and the standard version (using an independent samples *t*-test), finding no significant difference between the groups [*t*(321) = −1.68, *p* = 0.09].

The children coping strategy checklist (CCSC; α = 0.72–0.88; Ayers et al., 1996) is a 52-item self-report measure of coping strategies used in childhood and adolescence. For each item, the participant reports the frequency of the use of a specific coping strategy during stressful situations using a 4-point range of response (0, “never”; 1, “sometimes”; 2, “often”; 3, “most of the time”). The checklist involves four dimensions of coping: Active Coping Strategies, Avoidance Strategies, Distraction Strategies, Support Seeking Strategies. Example items include “you did something to make things better” (Active Coping), “you did something like videogames or a hobby” (Distraction), “you tried to stay away from the problem” (Avoidance), “you talked to someone who could help you solve the problem” (Support Seeking). Scores for each dimension are derived by taking the mean of the dimension, with higher scores indicating greater use of the strategy.

None of the measures were available in Lithuanian. Therefore, they were first independently translated in Lithuanian by a professional translator. The initial version of each measure was submitted to a group of English-speaking psychotherapists to ensure consistency, and the integrity of the measures was verified using back translation (Vallerand, 1989).

### Analyses

A preliminary check determined less than 5% missing data, indicating no need for imputation. Both Cronbach’s alpha showed a good level of internal consistency for the subscales of the TSCC: Anger (α = 0.83), Anxiety (α = 0.74), Depression (α = 0.79), Dissociation (α = 0.74), PTSD (α = 0.80), as well as the CYRM-R (α = 0.93), and the subscales of the CCSC: Active coping (α = 0.92), Avoidance (α = 0.72), Support Seeking (α = 0.83), and Distraction (α = 0.69). These results confirmed the suitability of the measures.

Pearson correlations were used to test the associations in hypotheses (1) and (2). To test hypothesis (3), a moderation analysis was run, following preliminary checks of the data to confirm normality, linearity, and an absence of significant outliers (Warner, 2012). A power analysis conducted using G^∗^Power (Version 3.1.9.4; Faul et al., 2007) indicated the sufficiency of the size of the sample for the moderation analysis^3^.

The moderation analysis was based on the code of model 1A from Stride et al. (2015), using ML as the estimator. Trauma symptoms were used as the outcome variables, which were regressed on (1) gender (male/female) and age (7–11 years/12–17 years), in order to control for their effects, (2) resilience scores, the four coping strategies (Active Coping, Support Seeking, Avoidance, and Distraction), and (3) four interaction terms (Resilience^∗^Active Coping, Resilience^∗^Support Seeking, Resilience^∗^Avoidance, and Resilience^∗^Distraction).

SPSS v25 (Ibm Corp, 2017) was used to run the correlation analyses, independent *t*-test, and tests of internal consistency. MPlus v8.3 (Muthén and Muthén, 2018) was used to run the moderation analysis.

## Results

### Hypothesis 1 and 2

Avoidant coping was significantly positively correlated with anxiety (*r* = 0.13, *p* < 0.05), depression (*r* = 0.19, *p* < 0.001), and PTSD symptoms (*r* = 0.25, *p* < 0.001). Support seeking coping was positively correlated with anxiety (*r* = 0.15, *p* < 0.001) and distraction coping was also positively correlated with anxiety (*r* = 0.12, *p* < 0.05) and also with depression (*r* = 0.12, *p* < 0.05). Social-ecological resilience was significantly negatively correlated with depression (*r* = −0.11, *p* < 0.05). No other significant associations were detected between resilience and trauma symptoms or coping strategies and trauma symptoms (Table 2).

**TABLE 2:** Correlations and descriptive statistics among study variables.

**Measure**	**Resilience**	**Active coping**	**Avoidant coping**	**Support seeking coping**	**Distraction coping**	**M (SD)**
Anxiety symptoms	−0.07	−0.03	0.13*	0.15**	0.12*	5.90 (4.11)
Depression symptoms	−0.11*	−0.05	0.19**	0.01	0.12*	6.31 (4.46)
PTSD symptoms	−0.06	−0.01	0.25**	0.05	0.10	9.03 (5.25)
*M (SD)*	31.60 (8.29)	1.43 (0.55)	1.29 (0.47)	1.27 (0.61)	1.27 (0.56)	

*p < 0.05; **p < 0.001; Pearson correlation coefficients are given for relationships (two-tailed).

### Hypothesis 3

**FIGURE 1:**
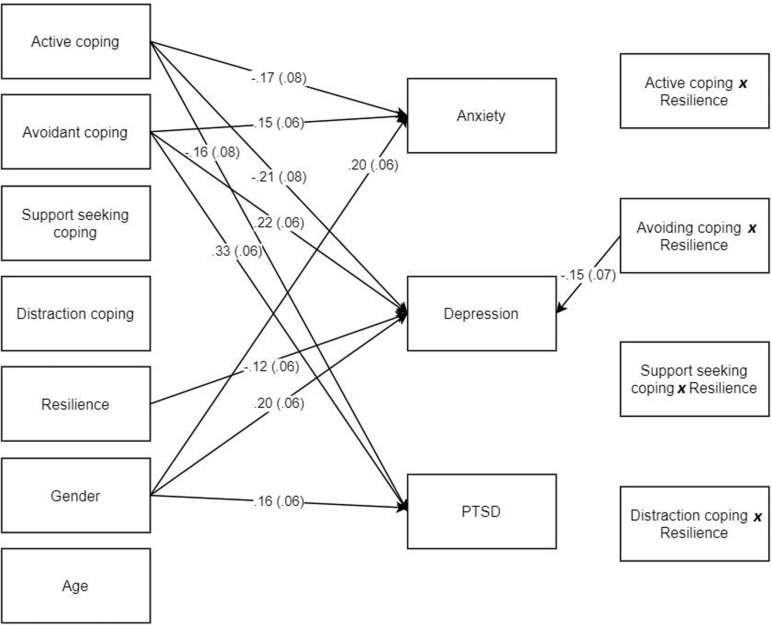
Moderation model of coping strategies and resilience predicting trauma outcomes. Only the significant pathways are shown.

In the moderation analysis, the predictors accounted for approximately 13% of the variance in scores for each kind of trauma (anxiety, depression, PTSD), each of which was significant (*p*s < 0.001) in this saturated model. Age was not associated with the trauma symptoms, while all the three types of the trauma symptoms were significantly higher for females than males (see Figure 1). In terms of main effects, we discovered active coping predicted lower levels of anxiety (β = −0.17, *p* = 0.024), depression (β = −0.21, *p* = 0.007), and PTSD (β = −0.16, *p* = 0.040), while avoidant coping predicted higher levels of anxiety (β = 0.15, *p* = 0.019), depression (β = 0.22, *p* < 0.001), and PTSD (β = 0.33, *p* < 0.001). No main effects were observed for support seeking or distraction coping. Social-ecological resilience was found to predict lower levels of depression (β = −0.12, *p* = 0.043), but not anxiety nor PTSD.

**FIGURE 2:**
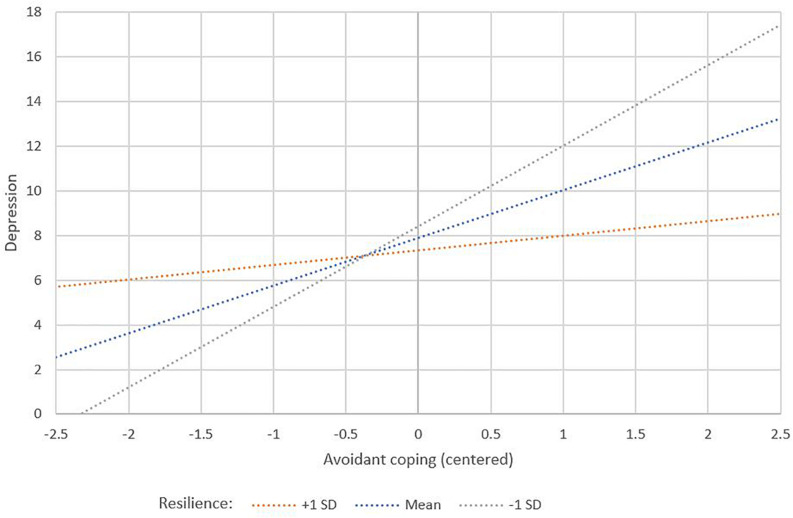
The interplay between active coping and resilience predicting depression symptoms.

Only one interaction was found to be significant: this was the interaction of social-ecological resilience^∗^avoidant coping for predicting depression (β = −0.15, *p* = 0.037) (Table 3). To aid in interpretation, this interaction was plotted using values of ±1 SD of the mean (as well as the mean) for social-ecological resilience (Aiken and West, 1991; Figure 2). The plot indicates that individuals with high avoidant coping and high resilience will have lower depression scores than those with high avoidant coping and low resilience. This relationship changes such that when an individual has a lower than average level of avoidant coping, those with higher resilience have higher depression scores compared to individuals lower resilience.

**TABLE 3:** Moderation path analysis results.

	**Estimate (β)**	**Standard error**	**95% CI of estimate**	
			**Lower**	**Upper**
Anxiety symptoms (*R*^2^ = 0.13**)				
Age (7–11 = 0, 12–17 = 1)	–0.09	0.06	–0.21	0.02
Gender (male = 0, female = 1)	0.20**	0.06	0.09	0.31
Resilience	–0.07	0.06	–0.18	0.05
Active coping	−0.17**	0.08	–0.32	-0.02
Avoidant coping	0.15**	0.06	0.02	0.27
Support seeking coping	0.13	0.07	0.00	0.27
Distraction coping	0.07	0.06	–0.05	0.20
Resilience*Active coping	0.06	0.09	–0.12	0.24
Resilience*Avoidant coping	–0.02	0.07	–0.16	0.13
Resilience*Support seeking coping	–0.07	0.07	–0.21	0.08
Resilience*Distraction coping	–0.08	0.07	–0.22	0.06
Depression symptoms (*R*^2^ = 0.14**)				
Age (7–11 = 0, 12–17 = 1)	0.05	0.06	–0.06	0.16
Gender (male = 0, female = 1)	0.20**	0.06	0.09	0.31
Resilience	−0.12*	0.06	–0.23	0.00
Active coping	−0.21*	0.08	–0.36	-0.06
Avoidant coping	0.22**	0.06	0.10	0.34
Support seeking coping	–0.04	0.07	–0.17	0.10
Distraction coping	0.11	0.06	–0.01	0.23
Resilience*Active coping	0.08	0.09	–0.10	0.26
Resilience*Avoidant coping	−0.15*	0.07	–0.29	-0.01
Resilience*Support seeking coping	0.00	0.07	–0.14	0.15
Resilience*Distraction coping	0.01	0.07	–0.14	0.15
PTSD symptoms (*R*^2^ = 0.13**)				
Age (7–11 = 0, 12–17 = 1)	–0.08	0.06	–0.20	0.03
Gender (male = 0, female = 1)	0.16*	0.06	0.05	0.27
Resilience	–0.04	0.06	–0.16	0.08
Active coping	−0.16*	0.08	–0.31	-0.01
Avoidant coping	0.33**	0.06	0.22	0.45
Support seeking coping	0.01	0.07	–0.13	0.15
Distraction coping	0.03	0.06	–0.10	0.15
Resilience*Active coping	–0.05	0.09	–0.23	0.13
Resilience*Avoidant coping	0.04	0.07	–0.10	0.19
Resilience*Support seeking coping	–0.04	0.07	–0.18	0.11
Resilience*Distraction coping	0.00	0.07	–0.14	0.14

β, standardized parameter estimates and bolded when significant; *p < 0.05; **p < 0.001.

## Discussion

To our knowledge, this study is the first to explore the interaction between specific coping strategies and social-ecological resilience in predicting trauma-related outcomes in children who have experienced adversity. Our study produced partial support for the hypotheses. First, there was mixed support for the hypothesis that coping strategies would be significantly associated with trauma-related symptoms. Support seeking coping positively correlated with anxiety, distraction coping strategies correlated positively with both anxiety and depression and avoidant coping strategies correlated positively with the three trauma-related outcomes considered (anxiety, depression, and PTSD). Instead, no correlation was found between active coping and any of the trauma-related symptoms. However, there was a significant main effect of both active coping and avoidant coping for each of the trauma symptoms. Also, relationships between support seeking and distraction coping with trauma symptoms disappeared in the moderation analysis. Therefore, when taking into account the impact of other variables such as gender, age, the presence or absence of other coping strategies and levels of social-ecological resilience, the relationship between many of the coping strategies and trauma-related outcomes appears to change. The crucial importance and fundamentally adaptive nature of active coping manifests, which may be due to individuals who are more capable of facing their fears, exhibiting low levels of denial, and exhibiting social competence, managing to actively cope with their stress and to show positive adjustment (Feder et al., 2011; Thompson et al., 2018). Meanwhile, the clear maladaptive nature of avoidance as a coping style appears, in line with previous studies exploring the impact of avoidance strategies on children’s trauma-related outcomes (Kraaij et al., 2003; Flett et al., 2012). Instead, the associations among other coping strategies (distraction and support-seeking), whose impact on mental health appeared to be more inconsistent in the literature (Compas et al., 2001), disappear.

These results are not entirely surprising given previous equivocal findings (Gil, 2005; Wright et al., 2007; Alim et al., 2008; Najdowski and Ullman, 2011), which indicate that although there might be evidence of an association between coping strategies and concurrent symptoms of distress and psychopathology, the causal role of coping in adjustment is much less clear (Compas et al., 2001). Indeed, the large number of studies showing non-significant effects of specific types of coping on mental health outcomes, suggests that the association between coping and trauma-related symptoms is inconsistent. Other factors need to be taken in consideration when testing the association of coping with psychological adjustment.

Support for the second hypothesis was also mixed. Social-ecological resilience was found to be negatively associated with depression symptoms, which is consistent with previous studies conducted with children exposed to trauma (Carbonell et al., 2002; Reivich et al., 2005; Anyan and Hjemdal, 2016; Poole et al., 2017), and with other research highlighting the protective roles of social resources on depressive outcomes and the related associations between a lack of perceived support and depressive outcomes in trauma-exposed individuals (Dumont and Provost, 1999; Schumm et al., 2006; Wilks, 2008; Tanigawa et al., 2011). However, no relationship was found between social-ecological resilience and anxiety or PTSD symptoms, contrary to other studies that have detected a relationship between resilience and similar mental health outcomes (Bensimon, 2012; Peltonen et al., 2014; Anyan and Hjemdal, 2016; Day and Kearney, 2016). This may be due to differences in the definition and measurement of resilience (Wu et al., 2015). For instance, none of the cited studies use definitions of resilience that appear to invoke a social-ecological perspective. Furthermore, their use of alternative measures such as the Connor Davidson Resilience Scale (CD-RISC) and the Resilience Scale for Adolescents (READ) means their definition of resilience is likely more heavily aligned with the use of psychological qualities. These differences in conceptualization and operationalization may account a discrepancy. However, other studies have noted the inconsistency of the association between resilience and mental health outcomes in cross-sectional studies (Siriwardhana et al., 2015).

Results of the third hypothesis showed that social-ecological resilience has a negatively moderating effect on the relationship between avoidant coping strategies and depression: that is, individuals with higher avoidant coping who also present good social-ecological resilience show lower depression symptoms compared to those with higher avoidant coping but low social-ecological resilience. Therefore, these external resources implicated in social-ecological resilience may provide a buffer against the commonly negative consequences of individuals with a propensity for avoidant coping.

Resilience is a dynamic process of interaction between risk and protective factors (Rutter, 2012). Therefore, the combination between different factors, rather than a single factor, can predict trauma-related outcomes in children exposed to adversity. In line with this, our results highlight the need to pay closer attention to interactions between factors, and, in particular, to the social context in which children encounter and try to cope with stress.

Several studies have underlined the buffering effects of social resources on depressive symptoms in children exposed to adversity (Yang et al., 2010; Tanigawa et al., 2011; Ungar, 2013). Our results showed that the impact of avoidant coping strategy on mental health outcomes differed depending on the resources available in their environment. This could be due to the fundamental role of social resources in providing coping assistance, for example, by helping to reinterpret situational demands, bolstering self-esteem, and sustaining a sense of mastery or competence through positive feedbacks and encouragement (Thoits, 1995). However, exploring the intervening mechanisms is a crucial next step, in order to understand the role of social environment in proposing effective adaption to stressors by assisting coping strategies (Nestmann and Hurrelmann, 1994; Thoits, 1995).

### Clinical Implications

The MHPSS professionals who design and implement interventions to enhance the likelihood of resilience among vulnerable children, should take in considerations the multiple interaction between the different factors that shape the resilience process. Indeed, resilience should not be conceived as the sum of individual’s resources, but rather as the interaction between risk and protective factors, between individual and social resources. Therefore, it is fundamental to explore the ways those factors concur in promoting children adjustment.

In particular, our results suggest the importance of considering both coping strategies and social resources of beneficiaries, as increasing resilience level may reflect a generalized positive effect on the child tendency to use functional coping strategies, while encouraging the use of specific coping strategies when facing certain trauma-related symptoms (i.e., depression) may increase the overall effect of resilience on the individual well-being. However, further studies investigating the malleability of coping and the ways in which the social context can facilitate effective coping in children and youth are needed in order to inform interventions for vulnerable children (Hooberman et al., 2010; Ng et al., 2012; Stratta et al., 2015).

In this perspective, when designing interventions, it appears fundamental assessing the resources available to the child and make social supports and formal services more available and accessible. This consideration lead to an important change of the locus of control in conceiving and designing intervention for vulnerable children: from what the child can do for him- or herself, to what the child’s broader community and service providers can and should do for the child (Obrist et al., 2010; Ungar, 2013). Further researches are needed to define what may be the most efficacious type of social support which matches the target individual’s needs.

### Limitations

Due to the particular nature of the population that we investigated, the number of children we could sample was restricted. Indeed, in Lithuania for long time violence against children has been considered a social taboo, and only on 2017, the year when the research took place, the Lithuanian Parliament finally passed amendments to the Law on the Fundamentals of Protection of the Rights of the Child (1996), prohibiting all corporal punishment of children. Hence, our results should be generalized with caution.

Furthermore, we only investigated the associations between variables in cross-sectional data, which do not allow to derive conclusions on the causal direction of the found associations. Studies collecting longitudinal data could clarify these directions. Future studies are also needed to explore the possible interactions between individual differences (e.g., temperament), coping strategies and social context and, in particular, to understand the mechanisms through which coping strategies, and social resources influence physical and mental.

In this study we only controlled for the effects of age and gender, finding that, according with previous literature (e.g., Olff et al., 2007), trauma-related symptoms were more serious for females than males, whereas age was not influential. A further future line of inquiry would be to investigate potential gender and age differences in the mechanisms through which coping strategies, and social resources may influence youth’s responses to the exposure to childhood adverse experiences.

### Conclusion

Although several studies highlight the association between coping strategies and concurrent symptoms of distress and psychopathology, our study suggests the role of coping in adjustment is complex. When controlling for other variables, such as sex, age, other coping strategies, and social-ecological resilience, only active coping was found to significantly predict each of the trauma-related symptoms. Our study also foregrounds the importance of considering social-ecological resilience and how this may interact with particular coping strategies; in particular, avoidant coping.

## Data Availability Statement

The datasets generated for this study are available on request to the corresponding author.

## Ethics Statement

The study was reviewed and approved by the Ethics Committee of the Department of Psychology of the Catholic University. The participants caregivers provided written informed consent to participate in this study.

## Author Contributions

FG and SC conceived and planned the research. FG wrote the manuscript with support of PJ, and developed the theory. PJ performed the analyses. FG and PJ discussed the results and contributed to the final manuscript. All authors contributed to the article and approved the submitted version.

## Conflict of Interest

The authors declare that the research was conducted in the absence of any commercial or financial relationships that could be construed as a potential conflict of interest.

## Footnotes

1 http://www.pvc.lt/en/

2 See https://cyrm.resilienceresearch.org

3 For a medium effect size effect size (0.15), α = 0.95, power = 0.8, and nine predictors, G^∗^Power indicated a minimum *n* of 114
